# Physics‐Informed Deep‐Learning For Elasticity: Forward, Inverse, and Mixed Problems

**DOI:** 10.1002/advs.202300439

**Published:** 2023-04-24

**Authors:** Chun‐Teh Chen, Grace X. Gu

**Affiliations:** ^1^ Department of Materials Science and Engineering University of California Berkeley CA 94720 USA; ^2^ Department of Mechanical Engineering University of California Berkeley CA 94720 USA

**Keywords:** artificial intelligence, computational methods, elastography, physics‐informed machine learning

## Abstract

Elastography is a medical imaging technique used to measure the elasticity of tissues by comparing ultrasound signals before and after a light compression. The lateral resolution of ultrasound is much inferior to the axial resolution. Current elastography methods generally require both axial and lateral displacement components, making them less effective for clinical applications. Additionally, these methods often rely on the assumption of material incompressibility, which can lead to inaccurate elasticity reconstruction as no materials are truly incompressible. To address these challenges, a new physics‐informed deep‐learning method for elastography is proposed. This new method integrates a displacement network and an elasticity network to reconstruct the Young's modulus field of a heterogeneous object based on only a measured axial displacement field. It also allows for the removal of the assumption of material incompressibility, enabling the reconstruction of both Young's modulus and Poisson's ratio fields simultaneously. The authors demonstrate that using multiple measurements can mitigate the potential error introduced by the “eggshell” effect, in which the presence of stiff material prevents the generation of strain in soft material. These improvements make this new method a valuable tool for a wide range of applications in medical imaging, materials characterization, and beyond.

## Graphical abstract

ElastNet learns the Young's modulus field of a heterogeneous object based on a measured displacement field. The predicted stress tensor is calculated by the encoded elastic constitutive relation based on the strain and Young's modulus. The training procedure minimizes the unbalanced forces with a physical constraint and updates the predicted Young's modulus using backpropagation.

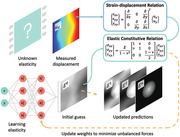

## 1 Introduction

Elastography is an emerging imaging modality to estimate the elasticity of biological tissues. Since its inception in 1991,^[^ ^1^ ^]^ the development of the field has produced a proliferation of methods and techniques from optimization strategies to clinical platforms.^[^ ^2^, ^3^, ^4^, ^5^, ^6^, ^7^, ^8^ ^]^ Strain‐based (quasi‐static) elastography is the first commercialized elastography technique. It measures local tissue strains by comparing ultrasound signals before and after a light compression. However, several limitations have prevented strain‐based elastography from being widely used in cancer diagnosis. As it is not possible to measure the stress distribution (field) of a body in vivo, the stress field is assumed to be uniform. Therefore, a measured strain field can be interpreted as a relative elasticity field based on Hooke's law. To ensure comparable stresses at different locations, the compression must be applied uniformly.^[^ ^2^, ^9^ ^]^ Consequently, strain‐based elastography is highly user‐dependent and requires experienced radiologists to obtain good‐quality elasticity images. Moreover, even if the compression is perfectly uniform, the stress field of a heterogeneous object is never uniform due to stress concentration.^[^ ^10^, ^11^ ^]^ These limitations lead to inaccurate elasticity reconstructions.

The limitations of strain‐based elastography have motivated researchers to develop more robust and accurate approaches using physics‐based modeling. These approaches, referred to as model‐based elastography, relax the assumption of stress uniformity and thus have the potential to provide more accurate predictions.^[^ ^3^, ^6^ ^]^ Model‐based elastography methods can be broadly grouped into two categories. The first is the direct approach, which treats measurements as coefficients in the partial differential equations (PDEs) for equilibrium.^[^ ^12^, ^13^, ^14^ ^]^ The second is the iterative approach, which is based on the finite element method (FEM) and minimizes the difference between measured and simulated displacements.^[^ ^15^, ^16^, ^17^, ^18^, ^19^ ^]^ The direct approach requires the strain and elasticity fields to be smooth (differentiable) and the elasticity on the boundary to be specified. Error from noise or inaccurate boundary values will propagate along the integration, reducing the accuracy of the solution.^[^ ^3^, ^12^ ^]^ The iterative approach requires significant computational resources and often depends on the choice of regularization and initiation strategies. In addition, two common disadvantages have limited the use of model‐based elastography in commercial systems. The first is the need for both axial and lateral displacement components. The lateral resolution of ultrasound exams is generally around 3 to 5 times worse than the axial resolution.^[^ ^20^ ^]^ Thus, a precise strain tensor cannot be calculated when one of the displacement components is inaccurate. The second is the assumption of material incompressibility. For an isotropic material, its response to mechanical loadings can be described by two independent elastic constants, Young's modulus and Poisson's ratio (or Lamé parameters).^[^ ^2^ ^]^ Most model‐based elastography methods assume that the material is incompressible (i.e., Poisson's ratio is 0.5) to reduce the complexity of the problem. Although biological tissues are typically composed of water and are often nearly incompressible,^[^ ^21^ ^]^ pathological tissues have been shown to have lower Poisson's ratios.^[^ ^22^ ^]^ Therefore, the assumption of material incompressibility may lead to inaccurate Young's modulus reconstructions.

Elastography problems are difficult to solve using conventional computational techniques due to the ill‐posed nature (i.e., lack of uniqueness or stability of solutions) of an inverse problem. A forward problem involves predicting the response of a physical system based on its present state and physical laws. In an inverse problem, the goal is to determine the present state of a physical system based on observations of its response. A classical forward problem in elasticity is to calculate the mechanical response of a given object subjected to various loadings. In this case, the elasticity field and boundary conditions are given. The mechanical response can be calculated using physics‐based methods (e.g., FEM). This forward problem can be inverted to create an inverse problem – that is, to reconstruct the elasticity field based on the mechanical response caused by excitations. This inverse problem, known as a coefficient inverse problem or inverse medium problem, is a longstanding challenge in elastography, and it is of great importance to many other applications, including materials characterization,^[^ ^23^ ^]^ biomedical engineering,^[^ ^24^ ^]^ and structural health monitoring.^[^ ^25^ ^]^

Recent advances in artificial intelligence and machine learning (ML) have provided an alternative approach to tackling complex scientific problems.^[^ ^26^, ^27^, ^28^, ^29^, ^30^ ^]^ Using machine learning to map one quantity of a physical system to another has been shown to be effective in solving a range of forward and inverse problems. For instance, ML models have been applied to solve inverse design problems of heterogeneous materials to achieve superior mechanical properties.^[^ ^31^, ^32^, ^33^ ^]^ Although mapping a displacement (or strain) field of an object to its elasticity field seems achievable using supervised learning models, such as generative adversarial networks,^[^ ^34^ ^]^ the generalization of these ML models is limited because the mapping is sorely based on data similarity. A fundamental issue is that a given elasticity field can produce an unlimited variety of displacement (or strain) fields depending on the boundary conditions. It is impractical to consider all possible boundary conditions when collecting training data. Consequently, predictions from ML models supervised by labeled data will not be accurate when the input (displacement or strain image) is not similar to (or a combination of) the training samples. In addition, these models operate as black boxes, and it is not clear how they arrive at their predictions because they do not incorporate physical laws in their training or use. Since many scientific problems can be described by PDEs, there is increasing interest in integrating physical laws into neural networks. Efforts to use neural networks to approximate solutions to PDEs go back to the early 1990s.^[^ ^35^, ^36^ ^]^ However, it is only recently that this new class of neural networks, called physics‐informed neural networks (PINNs), has been successfully employed to solve complex scientific problems.^[^ ^37^, ^38^, ^39^, ^40^ ^]^

In this work, we introduce a physics‐informed deep‐learning method for solving forward, inverse, and mixed elasticity problems. The goal of this work is to use deep neural networks (DNNs) guided by the theory of elasticity to learn the physical quantities of a heterogeneous object subjected to quasi‐static loading. These quantities include the displacement (axial and lateral) and elasticity (Young's modulus and Poisson's ratio) fields. This new method builds on our previous work, called ElastNet,^[^ ^41^ ^]^ but its capabilities are significantly enhanced by a new network architecture. We retain the name “ElastNet” for this new method, while the previous work is referred to as ElastNet‐2021.

## 2 Results and Discussion

### 2.1 Physics‐Informed Deep Learning for Elasticity

**Figure 1:**
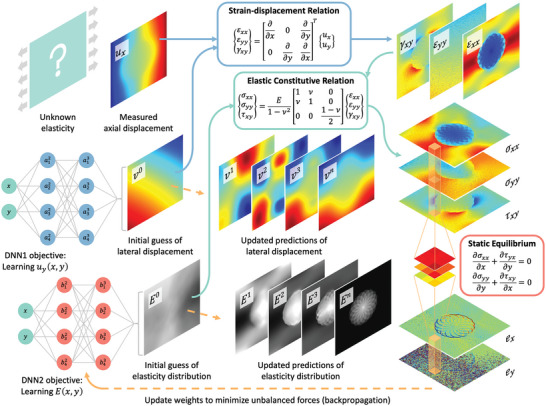
Framework of ElastNet. To solve a mixed elasticity problem, the displacement network (DNN1) takes the position at each material point and outputs its lateral displacement (*u_y_*). The elasticity network (DNN2) takes the position at each material point and outputs its Young's modulus (*E*). The predicted strain is calculated based on the measured axial displacement and predicted lateral displacement. The predicted stress tensor is calculated by the encoded elastic constitutive relation based on the strain and Young's modulus. The stress field is passed forward through a convolutional layer to calculate residual forces. The training procedure minimizes the norms of the residual forces with a physical constraint and updates the predicted lateral displacement and Young's modulus using backpropagation.

There is a need to improve ElastNet‐2021 in several respects to solve more complex elasticity imaging problems in real‐world applications. ElastNet‐2021 is limited to solving inverse elasticity problems with an unknown Young's modulus field. The new network architecture of ElastNet enables it to solve forward and mixed elasticity problems, which were not possible with ElastNet‐2021. This capability allows for the solution of all types of elasticity problems within the same deep‐learning framework. Additionally, ElastNet can solve inverse elasticity problems when both Young's modulus and Poisson's ratio fields are unknown. The framework of ElastNet is presented in **Figure** **1**. The network architecture, loss functions, and training procedure are provided in the Experimental Section. The innovation of ElastNet is that it integrates forward and inverse solvers by incorporating two DNNs – a displacement network (*f_d_*) and an elasticity network (*f_e_*). These two DNNs learn the displacement and elasticity fields of a given object, respectively. Technically, these two DNNs could be merged into a single network. However, we find that using two DNNs with different network architectures leads to more accurate predictions (as will be discussed later). Each DNN can predict up to two unknown physical quantities. Specifically, the displacement network can predict the axial and lateral components of the displacement field; the elasticity network can predict Young's modulus and Poisson's ratio of the elasticity field. However, the number of unknown physical quantities cannot be more than two since only two equilibrium conditions (i.e., the equilibrium in the *x*‐ and *y*‐directions) are available for solving the unknown quantities. Therefore, ElastNet can solve three types of elasticity problems. The first one is a forward elasticity problem, in which the elasticity field (Young's modulus and Poisson's ratio) of a given object and the boundary conditions are given. The goal is to determine the deformation (displacements or strains) of the object. The second one is an inverse elasticity problem, in which the displacement (or strain) field is given, and the objective is to determine the elasticity field of the object. The third one is a mixed elasticity problem, in which the displacement and elasticity fields are partially known, and the objective is to determine the unknown components.

The object of interest is assumed to be a thin plate in a plane stress state, with non‐zero stress components *σ*_*xx*_, *σ*_*yy*_, and *τ*_*xy*_. We will use a mixed elasticity problem to explain the physics and computations of ElastNet. This problem is motivated by quasi‐static ultrasound elastography, in which the axial component of a measured displacement field is much more accurate than the lateral component. With ElastNet, we only need to use the axial component for elasticity reconstruction and can disregard the lateral component. In this problem, we assume that the material is incompressible and the unknown elastic constant is Young's modulus. While it may not be of practical interest, one could disregard the axial component and use the lateral component for elasticity reconstruction, or assume that Young's modulus is known everywhere in the object and reconstruct the Poisson's ratio. In terms of implementation, these problems are equivalent. The object is discretized by uniformly distributed material points. External displacements are applied on the boundary to deform the object. The position (*p*) and axial displacement (*u_x_*) at each material point are collected as a dataset. The displacement network (*f_d_*) takes the position at each material point and outputs its lateral displacement (*u_y_* = *f_d_* (*p*)). The predicted strain tensor is calculated based on the measured axial displacement and predicted lateral displacement. The elasticity network (*f_e_*) takes the position at each material point and outputs its Young's modulus (*E* = *f_e_* (*p*)). The predicted stress tensor is calculated using the encoded elastic constitutive relation based on the strain and Young's modulus. Initially, since the lateral displacement and Young's modulus are generated by the DNNs with random weights, the predicted stress field is far away from satisfying the equilibrium conditions. To quantify how far from equilibrium the current predicted stress field is, it is passed forward through a convolutional layer to calculate unbalanced (residual) forces. The training procedure minimizes the norms of the residual forces with a physical constraint (see the Experimental Section for details) and updates the predicted lateral displacement and Young's modulus using backpropagation.

### 2.2 Mixed Elasticity Problems

**Figure 2:**
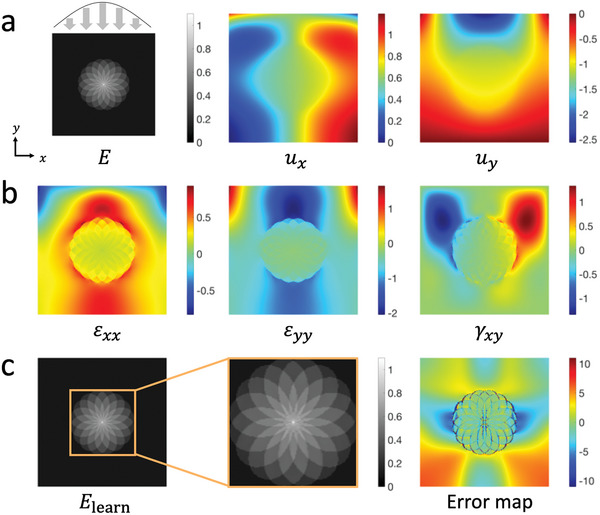
Rose model subjected to unevenly distributed displacements along the *y*‐direction. a) The Young's modulus field (MPa) and displacement field (mm). Axial displacements are applied on the top of the model and the distribution is a sinusoidal function with the largest amplitude at the center. b) The strain field (%). c) The learned Young's modulus field based on only the axial displacement field (*u_y_*) and relative error map (%). The MRE is 2.48%.

To evaluate the performance of ElastNet, we apply it to solve various forward, inverse, and mixed elasticity problems. In this work, the mean absolute error (MAE) is used to compare the performance of different activation functions on the same model. However, the MAE may not be an ideal measurement to compare the prediction accuracies between different models. A model with a larger elasticity field is likely to have a larger MAE. Therefore, the mean relative error (MRE) is used to compare the prediction accuracies between different models. We show that ElastNet can solve forward elasticity problems as accurately as FEM (Note S1 and Figures S1 and S2, Supporting Information). In this section, we focus on a more challenging problem in which the displacement and elasticity fields of a given object are partially known. A model with a rose‐shaped hard inclusion is considered, as shown in **Figure** **2**a. In this “rose” model, the Poisson's ratio is set to 0.5, and the Young's modulus is determined by the number of overlapping petals, ranging from 0.1 to 1.0 megapascal (MPa). To simulate manual compression using an ultrasound transducer, axial displacements are applied on the top of the model, with a sinusoidal distribution that has the largest amplitude at the center. The displacement and strain fields of the rose model are calculated using FEM and shown in Figure 2a,b, respectively.

Here, it is assumed that only the axial displacement field (*u_y_* in this case) is available. Therefore, the unknown physical quantities in this mixed elasticity problem are the Young's modulus and lateral displacement (*u_x_*) fields. The predicted Young's modulus field and relative error map are shown in Figure 2c, and the learning process is shown in Supplementary Movie S1. The results demonstrate that ElastNet provides an accurate prediction with an MRE of 2.48%. Since only the derivatives of the displacement field are used to solve the problem, an undetermined constant arises in the lateral displacement reconstruction. In certain applications, it may be of interest to determine the exact values of lateral displacements. In these cases, boundary conditions need to be specified. To demonstrate the versatility of ElastNet, we also consider another loading condition, in which the displacements are applied uniformly on the boundary, and observe a comparable MRE of 2.29% (Figure S3 and Movie S1, Supporting Information). Here, we consider an order of magnitude difference in the Young's modulus range based on the elastic properties of tissues in the human body.^[^ ^42^ ^]^ To evaluate the performance of ElastNet on materials with a wider range of Young's moduli, we also apply it to the rose model with a two‐orders of magnitude difference in the Young's modulus range. The prediction accuracy remains high with an MRE of 2.15% (Figure S4, Supporting Information). In addition to the rose model, 12 different models are used for a more comprehensive evaluation of ElastNet (Figures S5 and S6, and Table S1, Supporting Information). The MREs range from 0.70% to 4.42%, with an average of 1.96%.

### 2.3 Activation Functions

**Figure 3:**
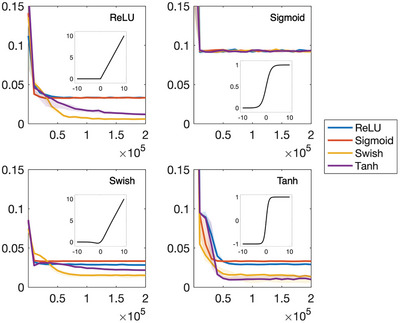
Performances of activation functions. In each sub‐figure, the activation function of the elasticity network (*σ*_*e*_) is the same and that of the displacement network (*σ*_*d*_) is varied. The *x*‐axis represents the number of training epochs and the *y*‐axis represents the MAE. Four activation functions, including the ReLU, sigmoid, swish, and tanh, are considered. The best performance is observed when *σ*_*e*_ is set to the ReLU and *σ*_*d*_ is set to the swish. The learning rate is 0.005.

The choice of activation function in deep learning can have a significant impact on the accuracy of the model's prediction. In physics‐informed deep learning, the derivatives of the activation function play an important role as physical laws are often encoded in the form of PDEs. While there is no one‐size‐fits‐all solution for selecting the best activation function, most physics‐informed deep‐learning methods have adopted a single DNN with one activation function. In contrast, ElastNet consists of two DNNs for predicting the displacement and elasticity fields, respectively. In this section, we evaluate the performance of four widely used activation functions, including the rectified linear unit (ReLU), standard logistic (sigmoid), swish, and hyperbolic tangent (tanh), in predicting different physical quantities using the rose model. We vary the activation function in the displacement network (*σ*_*d*_) while keeping the activation function in the elasticity network (*σ*_*e*_) the same. The results are presented in **Figure** **3** and Table S2, Supporting Information.

When *σ*_*e*_ is set to the ReLU, the lowest MAE is achieved when *σ*_*d*_ is set to the swish. The swish is a smooth and non‐monotonic function that has been found to perform better than other activation functions in image classification^[^ ^43^ ^]^ and physics‐informed deep‐learning problems.^[^ ^44^ ^]^ On the other hand, the worst performance is observed when *σ*_*d*_ is set to either the ReLU or sigmoid. The ReLU performs poorly in this case because the PDEs for equilibrium require the second derivatives of the displacement field, and the second derivative of the ReLU is zero. While ElastNet uses the finite difference method to calculate the derivatives of the PDEs, the second derivatives of the displacement field are rarely zero, even when the activation function is set to the ReLU. This lack of second‐order effects can lead to poor reconstruction of the Young's modulus field. The sigmoid also performs poorly because it suffers from vanishing gradient and non‐zero‐centered problems.^[^ ^45^ ^]^ Therefore, when *σ*_*e*_ is set to the sigmoid, ElastNet fails to reconstruct the Young's modulus field, regardless of which activation function is selected for *σ*_*d*_. When *σ*_*e*_ is set to either the swish or tanh, the lowest MAE is achieved when *σ*_*d*_ is set to the same activation function. Interestingly, the best performance is observed when *σ*_*e*_ is set to the ReLU and *σ*_*d*_ is set to the swish among all combinations considered. To confirm this finding, we also evaluate the activation functions with different learning rates and show that this combination outperforms using either the ReLU or swish alone (Figure S7, Supporting Information).

### 2.4 Inverse Elasticity Problems

**Figure 4:**
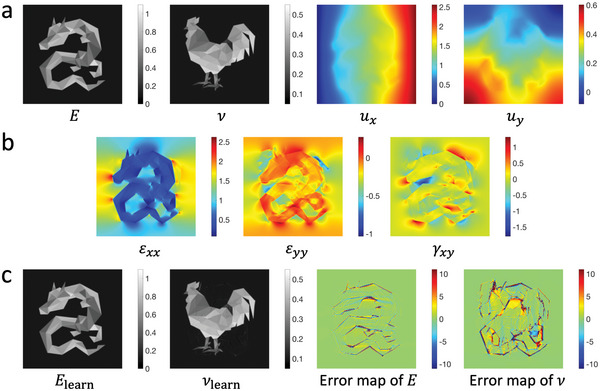
Dragon & rooster model subjected to displacements along the *x*‐direction. a) The Young's modulus field (MPa), Poisson's ratio field, and displacement field (mm). An average normal strain (*ε*_*xx*_) of 1% is introduced by the displacements applied along the *x*‐direction on the boundary. b) The strain field (%). c) The learned Young's modulus field, Poisson's ratio field, and relative error maps (%). The MRE of Young's modulus is 0.58% and the MRE of Poisson's ratio is 1.31%.

In this section, we examine inverse elasticity problems in which the displacement field of a given object is known and the objective is to determine the elasticity field. In ElastNet‐2021, we assumed that the material was incompressible. In this work, we remove the assumption and use two elastic constants to describe the elasticity of the material. To illustrate this, a model with a dragon‐shaped hard inclusion is considered, as shown in **Figure** **4**a. The Poisson's ratio is determined by a rooster‐shaped pattern. In this “dragon & rooster” model, the Young's modulus varies from 0.1 to 1.0 MPa. The Poisson's ratio varies from 0.1 to 0.5, as most materials have a Poisson's ratio in this range. Although some materials may have a zero Poisson's ratio, they are not considered because the MRE diverges when Poisson's ratio approaches zero. An average normal strain (*ε*_*xx*_) of 1% is introduced by the displacements applied along the *x*‐direction on the boundary. The displacement and strain fields are shown in Figure 4a,b, respectively. In this inverse elasticity problem, the unknown physical quantities are the Young's modulus and Poisson's ratio fields. The predictions and relative error maps are shown in Figure 4c, and the learning process is shown in Movie S2, Supporting Information. We demonstrate that ElastNet can simultaneously reconstruct these two elastic constants with high accuracy, with the MRE 0.58% for Young's modulus and 1.31% for Poisson's ratio.

### 2.5 Improving Predictions with Multiple Measurements

The performance of ElastNet, like other deep‐learning models, depends on factors such as the model capacity and learning algorithm. We also find that loading conditions can affect the prediction of ElastNet. For example, in the dragon & rooster model, when the displacements are applied along the *y*‐direction on the boundary, the MRE of Young's modulus is 0.50% and the MRE of the Poisson's ratio is 1.39% (Figure S8, Supporting Information). While the prediction accuracy is similar to that of the model subjected to displacements along the *x*‐direction, the relative error maps are slightly different. This is due to the “eggshell” effect, in which certain soft regions are confined by nearby hard inclusions, preventing their deformation.^[^ ^7^ ^]^ The intensity and location of the eggshell effect depend on the loading conditions, as the soft regions are not completely buried in the hard inclusions.

**Figure 5:**
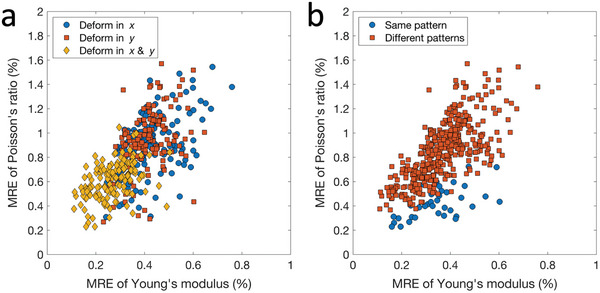
Improving predictions with multiple measurements. a) The results of 432 evaluations show that the MREs of the single‐loading cases (blue circles and red squares) are comparable and those of the duo‐loading cases (yellow diamonds) are considerably lower. b) The results shown in (a) are replotted based on whether the patterns of Young's modulus and Poisson's ratio are the same. The blue circles represent the results of the same patterns, and the red squares represent the results of different patterns. Lower MREs are achieved when the patterns are the same.

In this section, we explore the potential for improving prediction accuracy using multiple displacement fields obtained from different loading conditions. Results show that using two sets of displacement fields, one measured with displacements applied along the *x*‐direction and the other with displacements applied along the *y*‐direction leads to a lower MRE for both Young's modulus (0.36%) and Poisson's ratio (0.98%) compared to using a single displacement field. The dragon & rooster model is one of the 144 models used to evaluate the performance of ElastNet. The prediction accuracy for each model is evaluated with the single‐loading condition along the *x*‐direction, single‐loading condition along the *y*‐direction, and duo‐loading condition along the *x*‐ and *y*‐directions. The 432 evaluations are shown in **Figure** **5**a. In the figure, the MREs of the single‐loading cases are similar, while those of the duo‐loading cases are significantly lower. These results suggest that using multiple displacement fields can improve prediction accuracy. We also plot the results in Figure 5b, separated by whether the patterns of Young's modulus and Poisson's ratio are the same or different in the models. Results show that lower MREs are achieved when the patterns are the same. In practice, the patterns of the Young's modulus and Poisson's ratio fields of an object are often similar, as they both represent the distribution of different base materials. In these cases, ElastNet is expected to perform well.

### 2.6 Effect of Initiation Strategy on Prediction Accuracy

**Figure 6:**
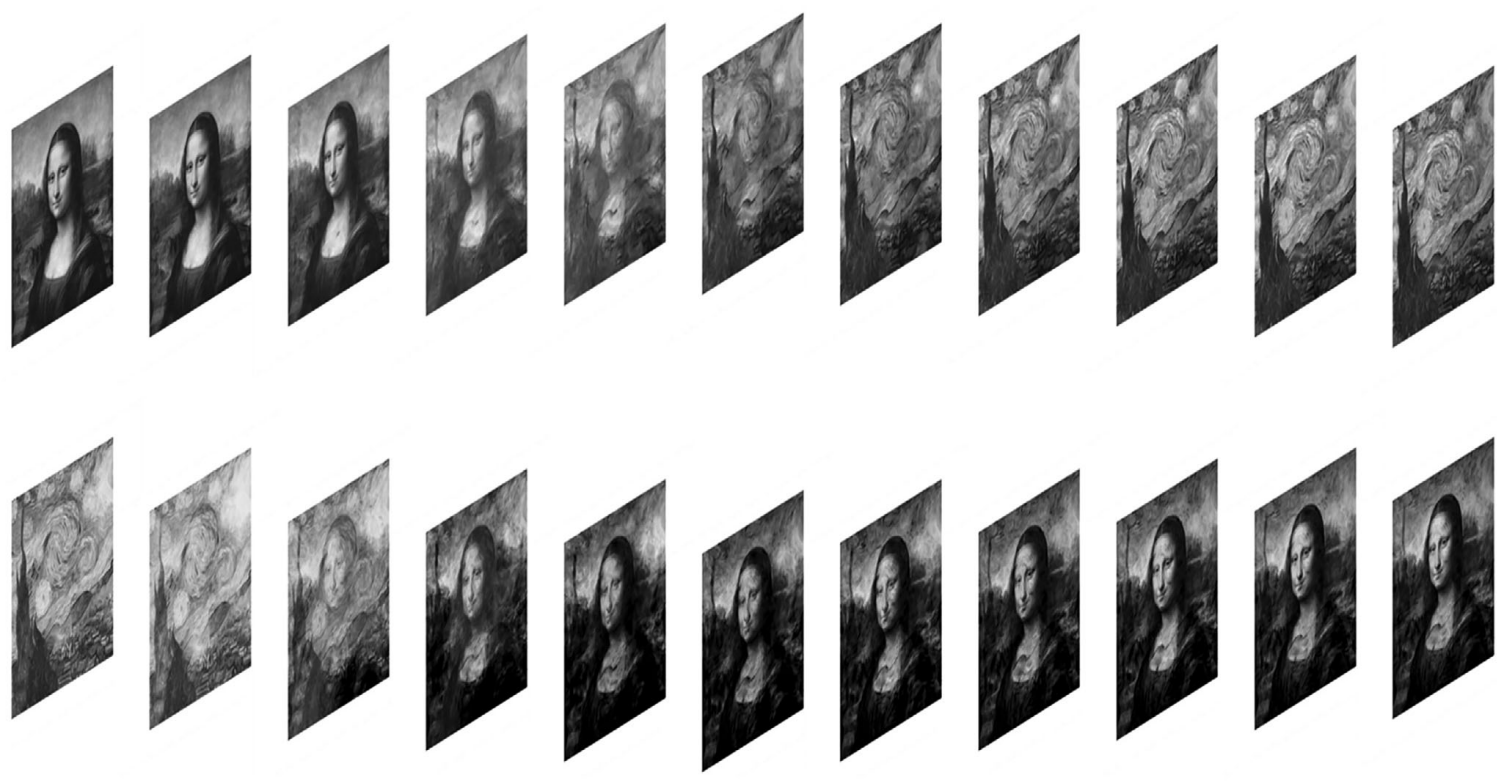
Learning process of the Starry Night & Mona Lisa model. The images show the intermediate predictions of the Young's modulus (upper images) and Poisson's ratio (lower images) fields during the first 100 training epochs. Instead of reinitializing the weights in ElastNet, the weights learned from the Mona Lisa & Starry Night model are adopted. Therefore, the initial Young's modulus field is close to the *Mona Lisa* and the initial Poisson's ratio field is close to the *Starry Night*. The final predictions after 800 000 training epochs and relative error maps are shown in Figure S11, Supporting Information.

ElastNet is an iterative approach, which requires an initial guess of the elasticity field to start with. This raises the question of whether the initiation strategy is important for ElastNet. To address this, we create a model with a complex elasticity field. The Young's modulus field is based on the *Mona Lisa* by Leonardo da Vinci, and the Poisson's ratio field is based on the *Starry Night* by Vincent van Gogh. In this “Mona Lisa & Starry Night” model, the Young's modulus varies from 0.1 to 1.0 MPa and the Poisson's ratio varies from 0.1 to 0.5. Two sets of displacement fields (Figure S9, Supporting Information) are used to reconstruct the elasticity field, and the learning process is presented in Movie S3, Supporting Information. We then create the “Starry Night & Mona Lisa” model (Figure S10, Supporting Information) by switching the patterns of the Young's modulus and Poisson's ratio fields of the previous model. Rather than reinitializing the weights in ElastNet, the weights learned from the previous model are reused. Thus, the initial Young's modulus field is similar to the *Mona Lisa* and the initial Poisson's ratio field is similar to the *Starry Night*. The learning process is presented in **Figure** **6** and Movie S4, Supporting Information. As with the previous model, the predictions and relative error maps show high accuracy, with the MRE 2.80% for Young's modulus and 3.39% for Poisson's ratio (Figure S11, Supporting Information). These results demonstrate that the initial elasticity field has no significant effect on prediction accuracy.

### 2.7 Related Work and Future Study

Several attempts have been made to develop physics‐informed deep‐learning methods for elastography, in addition to our previous work.^[^ ^41^ ^]^ Researchers have recently applied PINNs to discover the mechanical properties of tissue.^[^ ^46^ ^]^ However, their method does not predict displacement fields and therefore cannot solve the forward or mixed elasticity problems presented in this work. It also requires both axial and lateral displacement components, making it ineffective for ultrasound elastography. In addition, their method requires knowing stress distributions on all boundaries, which is not possible to measure in vivo. In contrast, ElastNet can achieve similar prediction accuracy without knowing stress distributions on the boundaries (Figure S12, Supporting Information). This unique feature of ElastNet also allows it to reconstruct only the elasticity field of the region of interest, rather than reconstructing the entire elasticity field defined by boundaries with known stress distributions (Figure S13, Supporting Information). Conventional iterative elastography methods based on FEM use the perturbation method to calculate numerical gradients for updating the prediction, while ElastNet uses backpropagation to calculate analytical gradients. As such, ElastNet is more accurate and computationally efficient than FEM‐based methods.

The assumption of linear elasticity is appropriate for this work as it is a common approximation used in many medical imaging applications. However, it is important to note that nonlinear elasticity can be observed in certain situations, especially when the applied strain is large. This is an area of active research and we believe that exploring the application of elastography methods to nonlinear elasticity problems would be an interesting direction for future work. In addition, it would be important to develop three‐demensional (3D) elastography methods as 3D ultrasounds become more popular. The framework of ElastNet can be applied to 3D systems with some minor modifications, such as changing the input space from a vector of two (*x* and *y*) to a vector of three (*x*, *y*, and *z*), changing the constitutive elasticity relation from 2D to 3D, and adjusting the equilibrium conditions from 2D to 3D. While ElastNet has been evaluated on simulated data and shown to have promising performance, future work will include experimental validation of the method using ultrasound equipment and phantoms. This will allow us to fully assess the performance of ElastNet in a real‐world setting and make any necessary adjustments.

## 3 Conclusion

In this work, we have demonstrated the potential of using physics‐informed deep learning to solve forward, inverse, and mixed elasticity problems. By integrating displacement and elasticity networks, our proposed method, ElastNet, can reconstruct the Young's modulus field of a heterogeneous object based on a measured axial displacement field without knowing stress distributions on boundaries. This is particularly beneficial for quasi‐static ultrasound elastography, as axial displacement measurements are more accurate than lateral ones and there is currently no technique for measuring stress distributions in vivo. In addition, ElastNet can remove the commonly‐made assumption of material incompressibility, allowing it to reconstruct both Young's modulus and Poisson's ratio fields simultaneously with high accuracy. This capability has potential applications in cancer diagnosis, as healthy and pathological tissues have distinct Young's moduli and Poisson's ratios, and in the characterization of general materials, as most materials are far from being incompressible. We have also shown that using multiple measurements can mitigate the potential error caused by the eggshell effect and improve prediction accuracy. While ElastNet requires an initial guess of the elasticity field to start with, we have demonstrated that the initial elasticity field has little effect on prediction accuracy. Finally, we have found that different activation functions may be needed for approximating different physical fields in PDEs. Our results highlight the potential of combining various activation functions to improve the prediction accuracy of physics‐informed deep‐learning methods for complex scientific problems.

## 4 Experimental Section

### Encoded Domain Knowledge of Elasticity

The learning process of ElastNet began with a displacement field, which could either be measured or predicted, depending on the problem at hand. The strain field was calculated from the displacement field, given by

(1) $$\varepsilon =\left\{\begin{array}{c} \varepsilon_{xx}\\ \varepsilon_{yy}\\ \gamma_{xy} \end{array}\right\}=\left\{\begin{array}{c} \frac{\partial u_{x}}{\partial x}\\ \frac{\partial u_{y}}{\partial y}\\ \frac{\partial u_{x}}{\partial y}+\frac{\partial u_{y}}{\partial x} \end{array}\right\}$$

where *ε* is the strain vector, and *u_x_* and *u_y_* are the horizontal and vertical components of the displacement, respectively. The derivatives of the displacement were calculated using a convolution operation (finite difference). The stress was calculated based on the constitutive elasticity relation for a linear elastic isotropic material in plane stress, given by

(2) $$\sigma =\left\{\begin{array}{c} \sigma_{xx}\\ \sigma_{yy}\\ \tau_{xy} \end{array}\right\}=\frac{E}{1-\nu^{2}}\left[\begin{array}{ccc} 1 & \nu & 0\\ \nu & 1 & 0\\ 0 & 0 & \left(1-\nu \right)/2 \end{array}\right]\left\{\begin{array}{c} \varepsilon_{xx}\\ \varepsilon_{yy}\\ \gamma_{xy} \end{array}\right\}$$

where *σ* is the stress vector, *E* is the Young's modulus, and *ν* is the Poisson's ratio. The equilibrium conditions were typically written in differential forms

(3) $$\begin{array}{c} \frac{\partial \sigma_{xx}}{\partial x}+\frac{\partial \tau_{yx}}{\partial y}=0\\ \frac{\partial \sigma_{yy}}{\partial y}+\frac{\partial \tau_{xy}}{\partial x}=0 \end{array}$$

ElastNet used a convolution operation to solve the PDEs. An object of interest was discretized by uniformly distributed material points. Here, the equilibrium of a sub‐region made up of 3 by 3 material points was considered. This sub‐region needed to satisfy the equilibrium conditions, given by

(4) $$\begin{array}{c} \sum_{a=1}^{3}\sum_{b=1}^{3}w_{xx}\left(a,b\right)\sigma_{xx}\left(a,b\right)+w_{yy}\left(a,b\right)\sigma_{yy}\left(a,b\right)+w_{xy}\left(a,b\right)\tau_{xy}\left(a,b\right)=0 \end{array}$$

where *w_xx_*, *w_yy_*, and *w_xy_* are 3 by 3 kernels for *σ*_*xx*_, *σ*_*yy*_, and *τ*_*xy*_, respectively. Two sets of kernels were used to describe the equilibrium conditions in the *x*‐ and *y*‐directions, respectively

(5) $$\begin{array}{c} \begin{array}{c} \sum F_{x}=0;\;w_{xx}=\left[\begin{array}{ccc} -1 & 0 & 1\\ -1 & 0 & 1\\ -1 & 0 & 1 \end{array}\right],\;w_{yy}=\left[\begin{array}{ccc} 0 & 0 & 0\\ 0 & 0 & 0\\ 0 & 0 & 0 \end{array}\right],\;w_{xy}=\left[\begin{array}{ccc} 1 & 1 & 1\\ 0 & 0 & 0\\ -1 & -1 & -1 \end{array}\right]\\ \sum F_{y}=0;\;w_{xx}=\left[\begin{array}{ccc} 0 & 0 & 0\\ 0 & 0 & 0\\ 0 & 0 & 0 \end{array}\right],\;w_{yy}=\left[\begin{array}{ccc} 1 & 1 & 1\\ 0 & 0 & 0\\ -1 & -1 & -1 \end{array}\right],\;w_{xy}=\left[\begin{array}{ccc} -1 & 0 & 1\\ -1 & 0 & 1\\ -1 & 0 & 1 \end{array}\right] \end{array} \end{array}$$

The residual force in each sub‐region was calculated by

(6) $$\begin{array}{ccc} e\left(i,j\right) & = & \sum_{a=1}^{3}\sum_{b=1}^{3}\left(w_{xx}\left(a,b\right)\sigma_{xx}\left(i+a-1,j+b-1\right)\right.\\ & & \left.+\;w_{yy}\left(a,b\right)\sigma_{yy}\left(i+a-1,j+b-1\right)\right.\\ & & \left.+\;w_{xy}\left(a,b\right)\tau_{xy}\left(i+a-1,j+b-1\right)\right)ht \end{array}$$

where *h* is the sides of a subregion and *t* is the thickness.

### Neural Network Architecture

ElastNet consisted of two DNNs: a displacement network and an elasticity network. Each DNN was composed of 16 fully connected hidden layers, each with 192 (the Mona Lisa & Starry Night models) or 128 (the other models) neurons. The activation functions in the displacement and elasticity networks were the swish and ReLU, respectively. The output of the elasticity network utilized the sigmoid function to squash the output for Poisson's ratio to be within the range of 0 and 0.5. The Young's modulus, however, was not constrained to any specific range. The input of both DNNs was a vector of two variables (*x*, *y*) representing the position of a material point. The output of the displacement network could include the axial and lateral displacements of the point; the output of the elasticity network could include the Young's modulus and Poisson's ratio of the point. For forward elasticity problems, where the elasticity field was given, the elasticity network was omitted. Similarly, for inverse elasticity problems, where the displacement field was given, the displacement network was omitted. In mixed elasticity problems, either the axial or lateral displacement field was predicted by the displacement network and either the Young's modulus or Poisson's ratio field was predicted by the elasticity network.

### Forward Elasticity Problems

In forward elasticity problems, the loss function consisted of three terms: the residual forces (*L_r_*), geometric boundary conditions (*L_u_*), and force boundary conditions (*L_f_*). The total loss was calculated as the weighted sum of these three terms, defined as

(7) $$L=w_{r}L_{r}+w_{u}L_{u}+w_{f}L_{f}$$

where *w_r_*, *w_u_*, and *w_f_* are the weights for these three terms. The loss of residual forces was calculated by the normalized MAE, defined as

(8) $$L_{r}=\frac{1}{p^{2}}\sum_{i}^{p}\sum_{j}^{p}\frac{\left|e\left(i,j\right)\right|}{{\hat{E}}_{\mathrm{pred}}\left(i,j\right)}$$

where *p* is the dimension of the residual force map, *e* is the residual force in a sub‐region, and ${\hat{E}}_{\mathrm{pred}}$ is the sum of the predicted Young's modulus values in a sub‐region, defined as

(9) $${\hat{E}}_{\mathrm{pred}}\left(i,j\right)=\sum_{a=1}^{3}\sum_{b=1}^{3}E_{\mathrm{pred}}\left(i+a-1,j+b-1\right)$$

The calculation was done by sliding a 3 by 3 kernel of all ones. The loss of geometric boundary conditions was calculated by the MAE, defined as

(10) $$L_{u}=\frac{1}{N_{u}}\sum_{i=1}^{N_{u}}\left|u_{i}-u_{\mathrm{pred}}\left(x_{i},y_{i}\right)\right|$$

where *u* is the displacement specified over a portion of the boundary, *u_pred_* is the predicted displacement, and *N_u_* is the number of points on the portion of the boundary. The loss of force boundary conditions was calculated by the MAE, defined as

(11) $$L_{f}=\frac{1}{N_{t}}\sum_{i=1}^{N_{t}}\left|t_{i}-t_{\mathrm{pred}}\left(x_{i},y_{i}\right)\right|$$

where *t* is the traction specified over a portion of the boundary, *t_pred_* is the predicted traction, and *N_f_* is the number of points on the portion of the boundary.

### Inverse Elasticity Problems

In inverse elasticity problems, no unique Young's modulus field could be determined from a displacement (or strain) field alone. A physical constraint based on the mean elasticity was imposed to obtain a unique Young's modulus field. In practice, when the mean elasticity was not available, an arbitrary value (larger than zero) could be specified and a relative Young's modulus field could be obtained. More discussions on the physical constraint can be found in the previous work.^[^ ^41^ ^]^ In inverse elasticity problems, the loss function consisted of two terms: the residual forces (*L_r_*) and physical constraint based on the mean elasticity (*L_e_*). The total loss was calculated as the weighted sum of these two terms, defined as

(12) $$L=w_{r}L_{r}+w_{e}L_{e}$$

where *w_r_* and *w_e_* are the weights for these two terms. The loss of the mean elasticity was calculated as the difference between the predicted and actual mean elasticity values, over the entire elasticity image

(13) $$L_{e}=\frac{1}{q^{2}}\left|\sum_{i=1}^{q}\sum_{j=1}^{q}E_{\mathrm{pred}}\left(i,j\right)-\sum_{i=1}^{q}\sum_{j=1}^{q}E\left(i,j\right)\right|$$

where *q* is the dimension of the elasticity image.

### Mixed Elasticity Problems

In mixed elasticity problems, the displacement network was used to predict either the lateral or axial displacement field. However, as only the derivatives of the displacement field were used in solving the problem, an undetermined constant arose in the displacement field reconstruction. A physical constraint based on the mean displacement was imposed to obtain a unique displacement field. Additionally, a physical constraint based on the mean elasticity was also imposed. The loss function consisted of three terms: the residual forces (*L_r_*), physical constraint based on the mean elasticity (*L_e_*), and physical constraint based on the mean displacement (*L_d_*). The total loss was calculated as the weighted sum of these three terms, defined as

(14) $$L=w_{r}L_{r}+w_{e}L_{e}+w_{d}L_{d}$$

where *w_r_*, *w_e_*, and *w_d_* are the weights for these three terms. The loss of the mean displacement was defined as

(15) $$L_{d}=\frac{1}{r^{2}}\left|\sum_{i=1}^{r}\sum_{j=1}^{r}u_{\mathrm{pred}}\left(i,j\right)\right|$$

where *r* is the dimension of the displacement image and *u_pred_* is the predicted displacement. This term forced the mean of the predicted displacement field to be zero, thus allowing the learning algorithm to find a unique displacement field. One could change this term to force the mean of the predicted displacement field to be any value. Doing so would not change the prediction of the Young's modulus field.

### Neural Network Training

The loss was minimized using the Adam optimizer.^[^ ^47^ ^]^ The training process was implemented using TensorFlow.^[^ ^48^ ^]^ To better evaluate the performance of ElastNet, it was trained 10 times with random initial weights for each model. The final prediction was obtained by averaging the five predictions with the lowest loss values. For the Mona Lisa & Starry Night models, the training process was carried out for 800 000 epochs, while for the other models, it was done for 200 000 epochs.

### Error Measurements

The error during the learning process was quantified by the MAE, defined as

(16) $$MAE=\frac{1}{q^{2}}\sum_{i}^{q}\sum_{j}^{q}\left|E_{\mathrm{pred}}\left(i,j\right)-E\left(i,j\right)\right|$$

The MRE was used to compare the prediction accuracies between different models, defined as:

(17) $$MRE=\frac{100}{q}\sum_{i}^{q}\sum_{j}^{q}\left|\left(E_{\mathrm{pred}}\left(i,j\right)-E\left(i,j\right)\right)/E\left(i,j\right)\right|$$

### Finite Element Analysis

The models in this work were discretized by four‐node quadrilateral elements. The Mona Lisa model (Figure S13, Supporting Information) had a mesh of 512 by 512, while the other models had a mesh of 256 by 256. The boundary conditions for the models are described in Figure S1, Supporting Information. The details of the finite element analysis can be found in the previous work.^[^ ^41^ ^]^ The rose model was created based on an equation, defined as

(18) $$r=\sin\left(\frac{8\theta }{5}\right)$$

where *r* is the radial distance from the origin and *θ* is the counterclockwise angle from the *x*‐axis. Additionally, the 12 Chinese zodiac models (Figure S5, Supporting Information) were created based on an image from hudasaktian/Shutterstock.com. These 12 Chinese zodiac patterns were used to generate 144 models, with the Young's modulus and Poisson's ratio fields based on one of the 12 patterns respectively. Among these 144 models, 12 of them had the same pattern for the Young's modulus and Poisson's ratio fields, while the remaining models had different patterns.

## Conflict of Interest

The authors declare no conflict of interest.

## Author Contributions

C.‐T.C. and G.X.G. conceived the idea. C.‐T.C. designed the theory and modeling approach, implemented the simulations, and analyzed the data. C.‐T.C. wrote the manuscript with valuable input from G.X.G.

## Supporting information

Supporting Information

Supplemental Movie 1

Supplemental Movie 2

Supplemental Movie 3

Supplemental Movie 4

## Data Availability

The data that support the findings of this study are available in the supplementary material of this article.
