# Supplementary material for: Physics‐Informed Deep‐Learning For Elasticity: Forward, Inverse, and Mixed Problems
Source: Adv Sci (Weinh). 2023 Apr 24;10(18):2300439. doi: 10.1002/advs.202300439 (PMC10288249; doi:10.1002/advs.202300439)
Supplement: Supplementary file 1 — Supporting Information [file ADVS-10-2300439-s004.pdf]

ADVANCED SCIENCE
Open Access

Supporting Information

for *Adv. Sci.*, DOI 10.1002/advs.202300439

Physics-Informed Deep-Learning For Elasticity: Forward, Inverse, and Mixed Problems

*Chun-Teh Chen and Grace X. Gu**

Supplementary Information

Physics-informed deep-learning for elasticity: forward, inverse, and mixed problems

Chen and Gu

Supplementary Figures

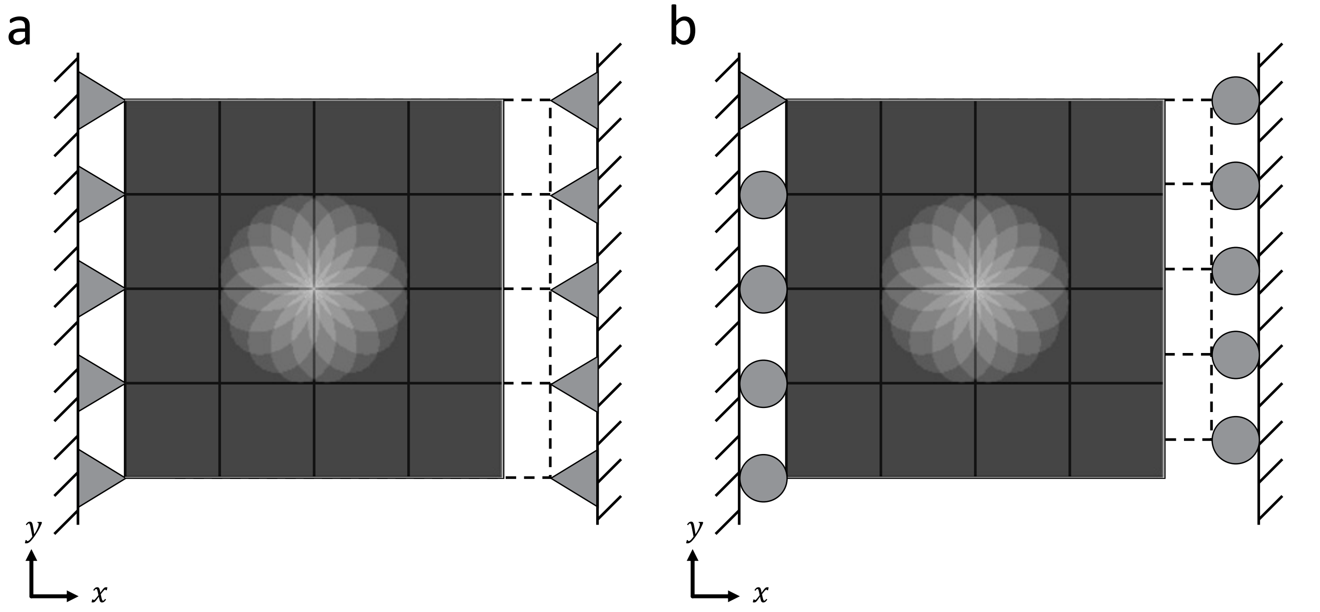

Supplementary Figure 1: Boundary conditions adopted in this work. (a) The boundary conditions adopted in the forward elasticity problem. The top and bottom boundaries are free, and the left boundary is fixed. External displacements along the *x*-direction are applied on the right boundary. Movements are not allowed along the vertical direction (*y*-direction) on the right boundary. The applied displacements are 1% of the model length. Therefore, an average normal strain along the *x*-direction (ε_{xx}) of 1% is generated. (b) The boundary conditions adopted in the inverse and mixed elasticity problems. The difference between (a) and (b) is that the left boundary is not fully fixed. Movements are not allowed along the horizontal direction (*x*-direction) but are allowed along the vertical direction (*y*-direction). Movements are also allowed along the vertical direction (*y*-direction) on the right boundary.

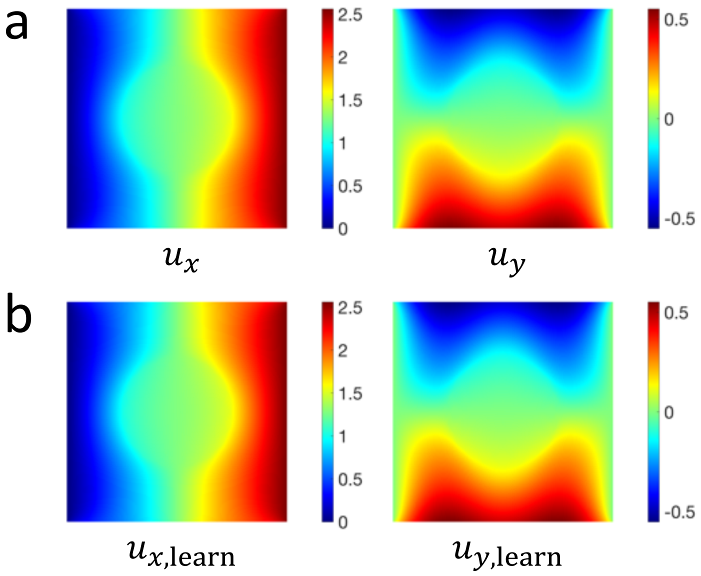

Supplementary Figure 2: Comparison of displacement fields generated using FEM and ElastNet. (a) The displacement field calculated using FEM. (b) The displacement field predicted using ElastNet. The R^2 values of the horizontal (u_x) and vertical (u_y) components of the displacement field are 0.9999 and 0.9996, respectively. The rose model is used for the comparison.

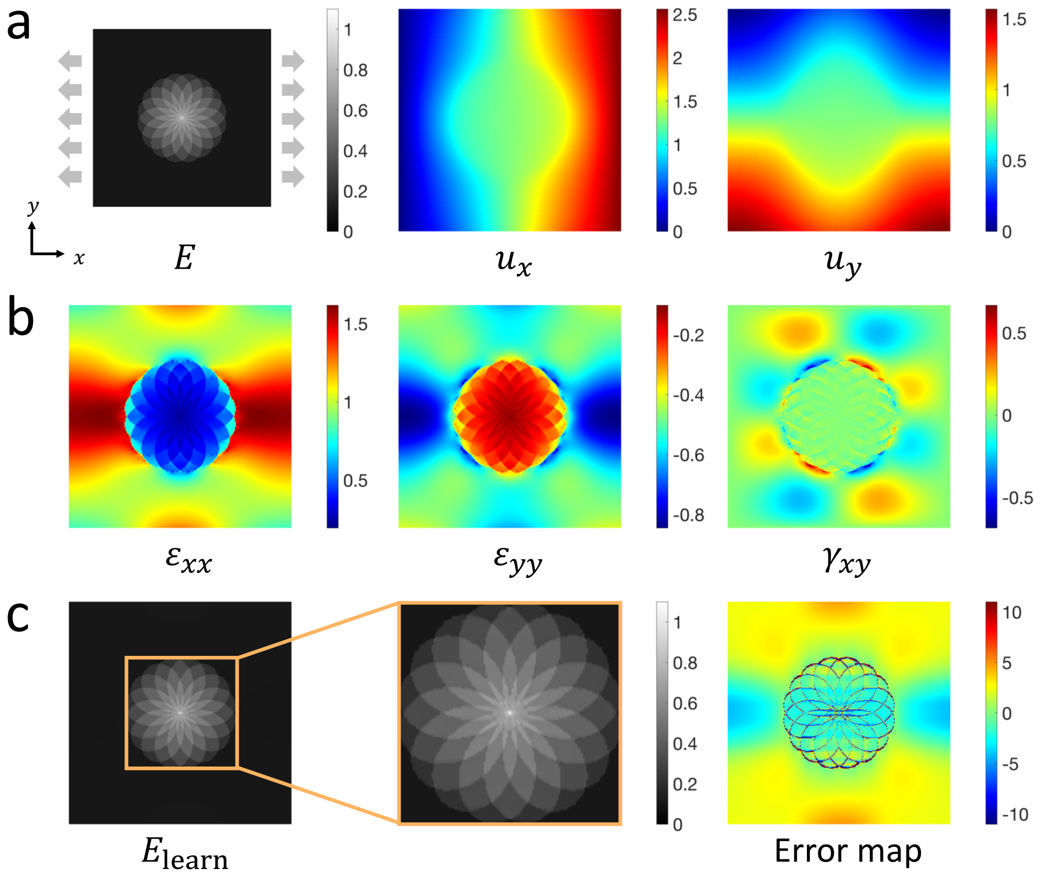

Supplementary Figure 3: Rose model subjected to displacements along the *x*-direction. (a) The Young's modulus field (MPa) and displacement field (mm). An average normal strain (ε_{xx}) of 1% is introduced by the displacements applied along the *x*-direction on the boundary. (b) The strain field (%). (c) The learned Young's modulus field based on only the axial displacement field (u_x) and relative error map (%). The MRE is 2.29%.

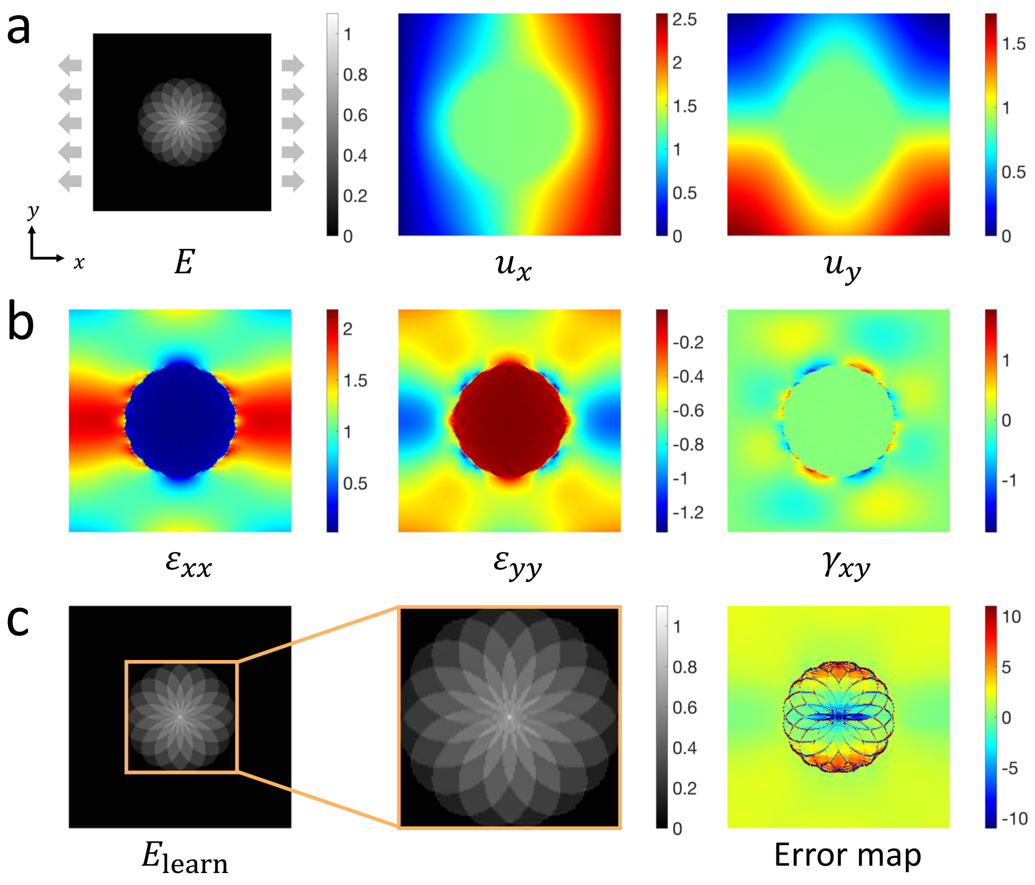

Supplementary Figure 4: Rose model with the Young's modulus range from 0.01 to 1.0 MPa subjected to displacements along the *x*-direction. (a) The Young's modulus field (MPa) and displacement field (mm). An average normal strain (ε_{xx}) of 1% is introduced by the displacements applied along the *x*-direction on the boundary. (b) The strain field (%). (c) The learned Young's modulus field based on only the axial displacement field (u_x) and relative error map (%). The MRE is 2.15%.

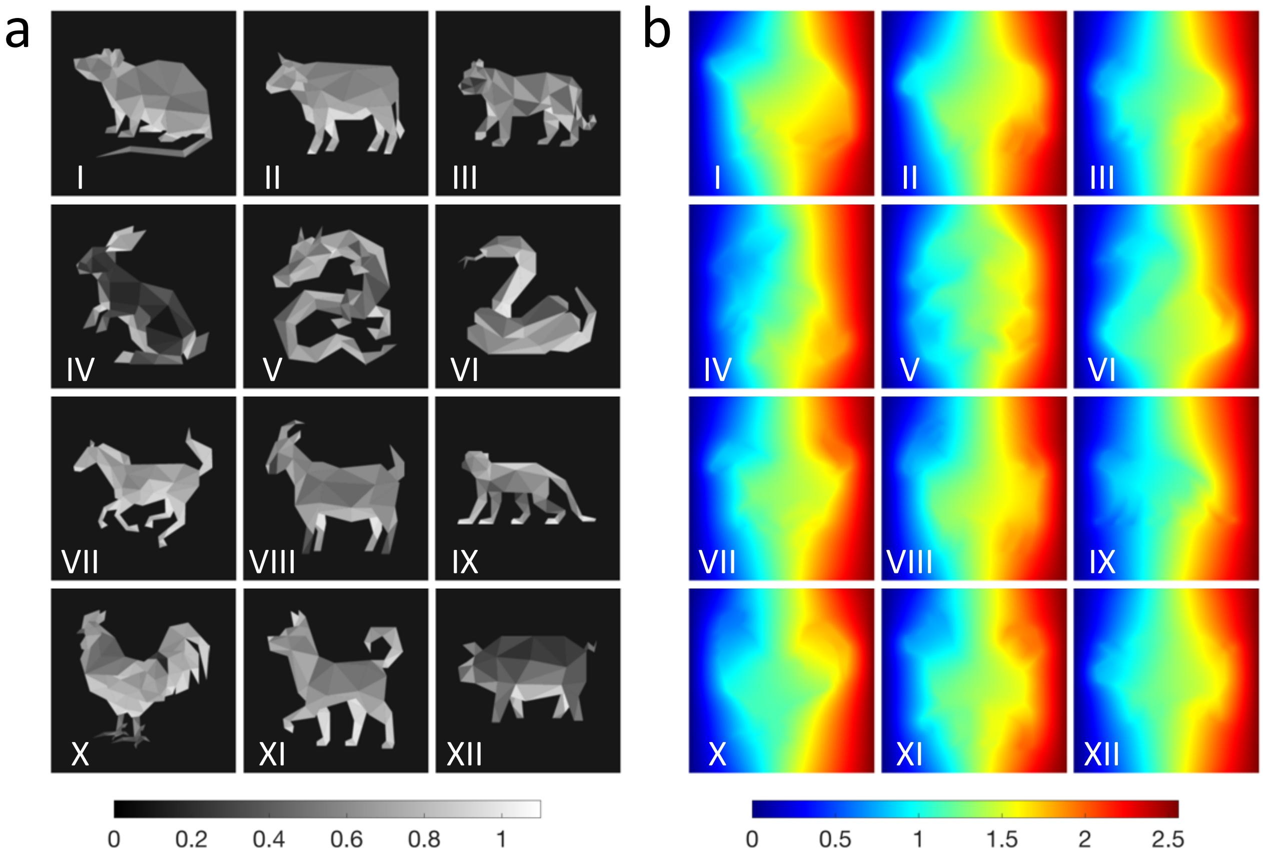

Supplementary Figure 5: Chinese zodiac models subjected to displacements along the *x*-direction. (a) The Young's modulus fields (MPa). (b) The axial displacement fields (mm). An average normal strain (ε_{xx}) of 1% is introduced by the displacements applied along the *x*-direction on the boundary.

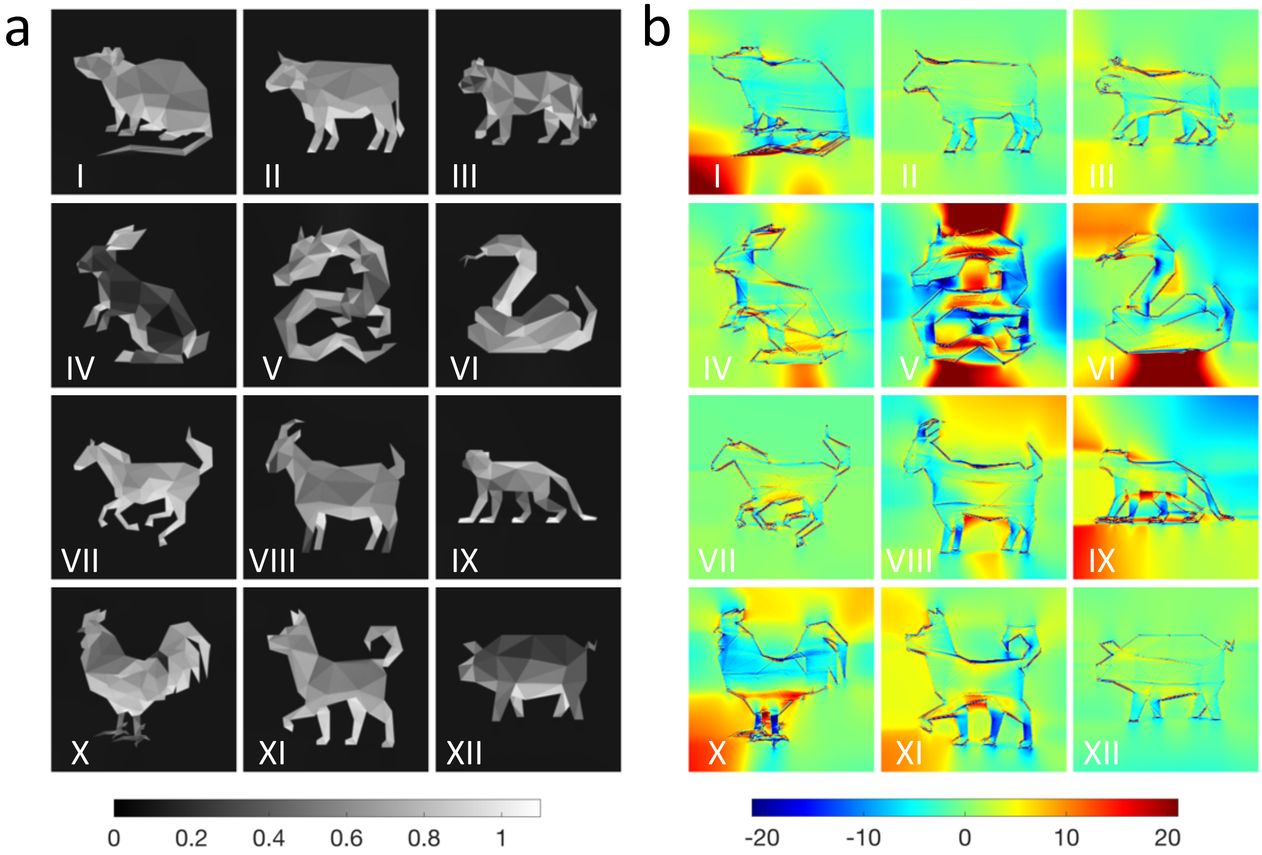

Supplementary Figure 6: ElastNet predictions for the Chinese zodiac models. (a) The learned Young's modulus fields (MPa) based on only the axial displacement fields (u_x). (b) The relative error maps (%). The average MRE is 1.96%.

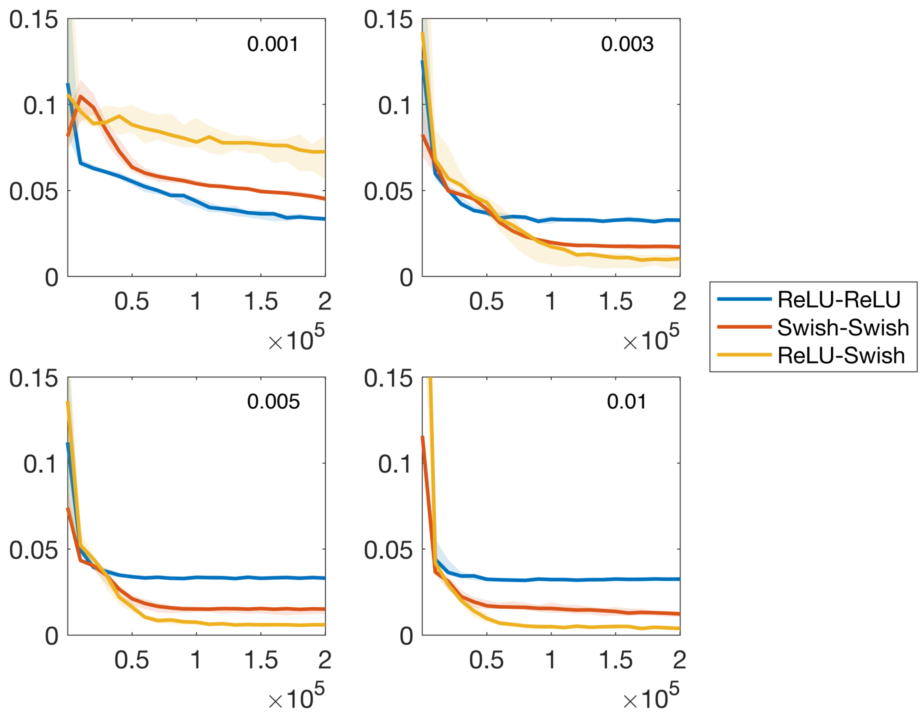

Supplementary Figure 7: Performances of activation functions with different learning rates. In each sub-figure, the learning rate is the same and the combination of activation functions is varied. The *x*-axis represents the number of training epochs and the *y*-axis represents the MAE. Four learning rates, including 0.001, 0.003, 0.005 and 0.01, are considered. The best performance is observed when σ_e is set to the ReLU and σ_d is set to the swish (yellow curve).

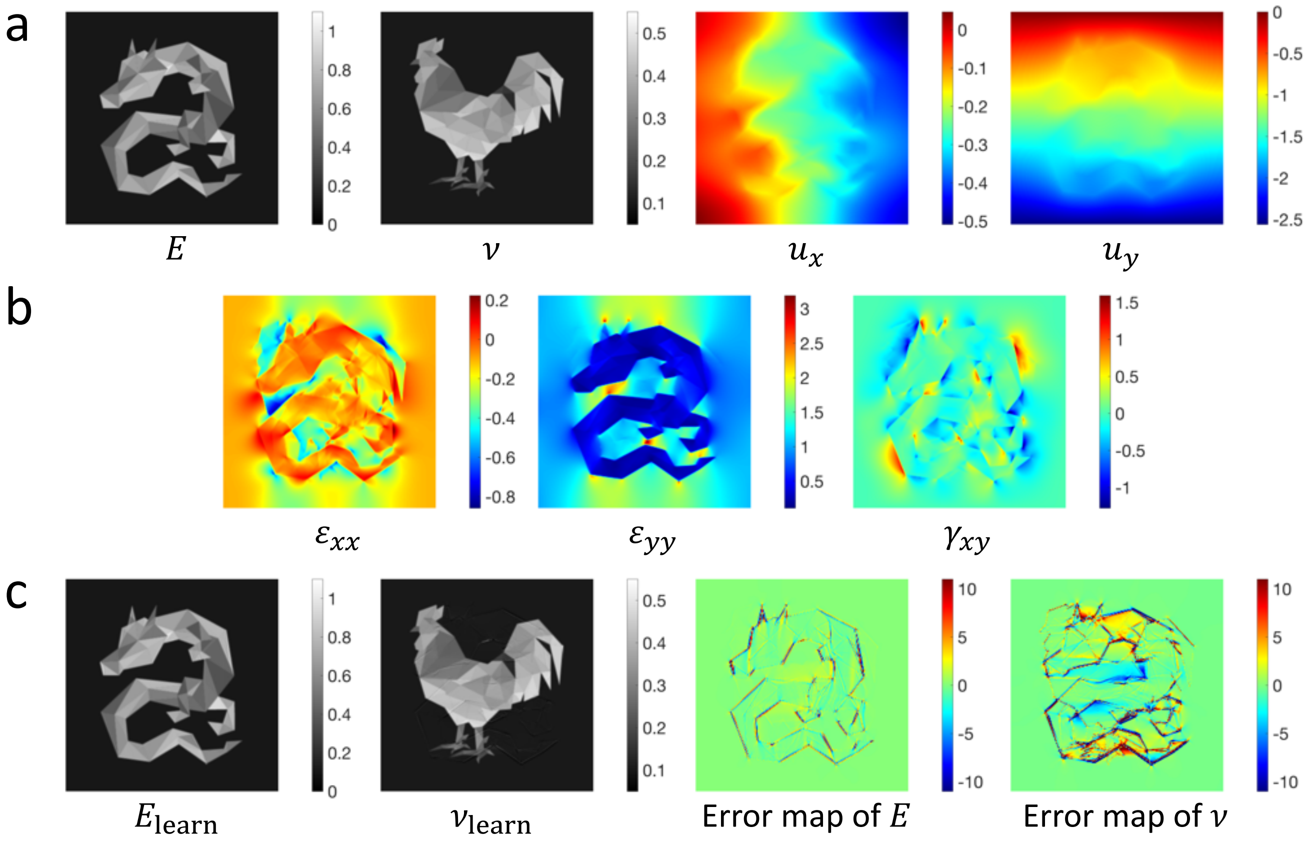

Supplementary Figure 8: Dragon & rooster model subjected to displacements along the *y*-direction. (a) The Young's modulus field (MPa), Poisson's ratio field, and displacement field (mm). An average normal strain (ε_{yy}) of 1% is introduced by the displacements applied along the *y*-direction on the boundary. (b) The strain field (%). (c) The learned Young's modulus field, Poisson's ratio field, and relative error maps (%). The MRE of the Young's modulus is 0.50% and the MRE of the Poisson's ratio is 1.39%.

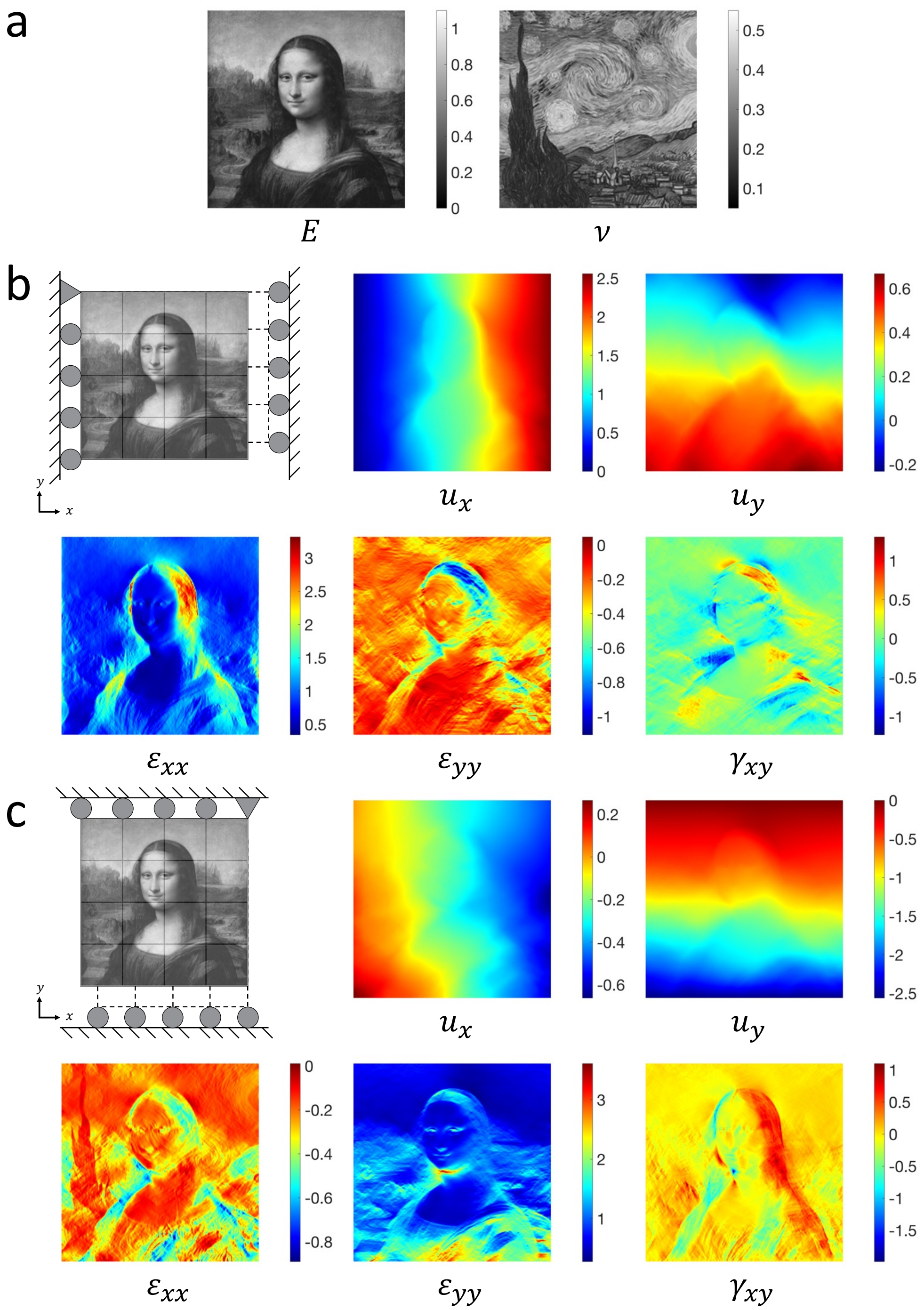

Supplementary Figure 9: Mona Lisa & Starry Night model subjected to displacements along the *x*-direction and *y*-direction. (a) The Young's modulus (MPa) and Poisson's ratio fields. The displacement (mm) and strain (%) fields when the model is subjected to displacements along the (b) *x*-direction and (c) *y*-direction, respectively.

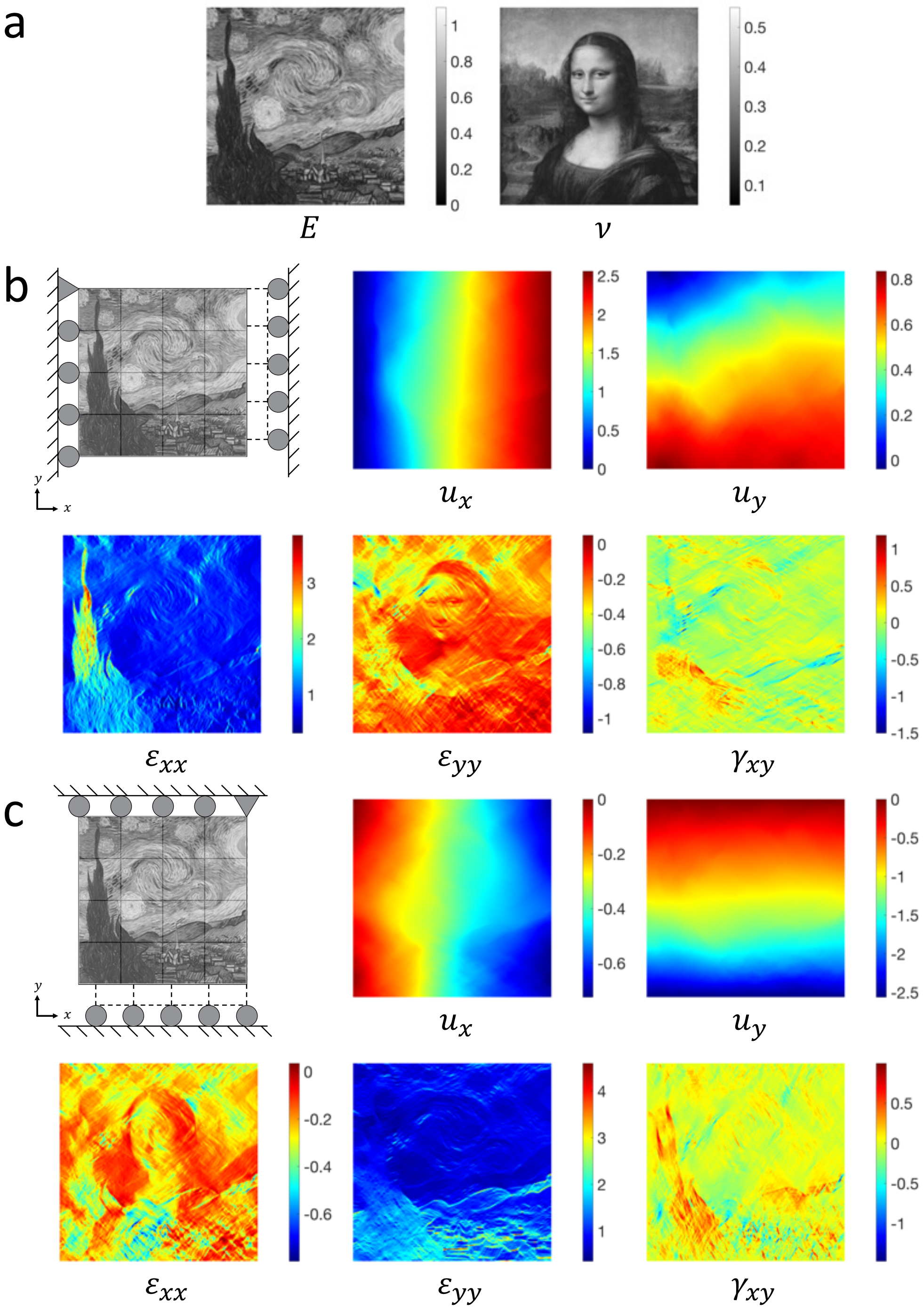

Supplementary Figure 10: Starry Night & Mona Lisa model subjected to displacements along the *x*-direction and *y*-direction. (a) The Young's modulus (MPa) and Poisson's ratio fields. The displacement (mm) and strain (%) fields when the model is subjected to displacements along the (b) *x*-direction and (c) *y*-direction, respectively.

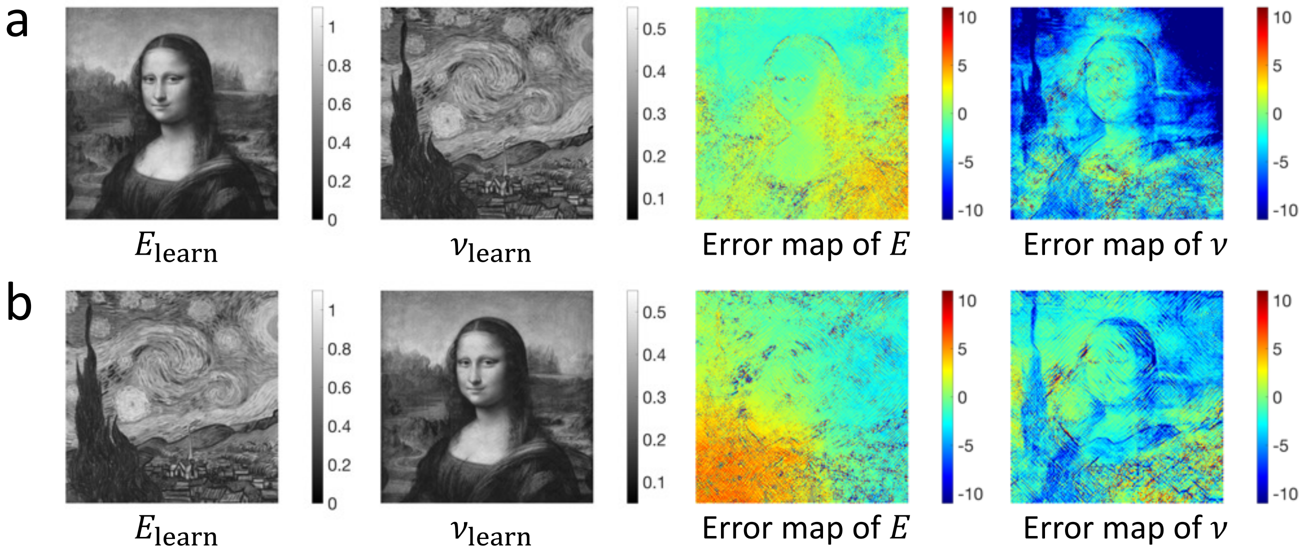

Supplementary Figure 11: ElastNet predictions for the Mona Lisa & Starry Night models. (a) The learned Young's modulus field (MPa), Poisson's ratio field, and relative error maps (%) for the Mona Lisa & Starry Night model. The MRE of the Young's modulus is 1.89% and the MRE of the Poisson's ratio is 6.08%. (b) The learned Young's modulus field, Poisson's ratio field, and relative error maps for the Starry Night & Mona Lisa model. Instead of reinitializing the weights in ElastNet, the weights learned from the Mona Lisa & Starry Night model are reused. The MRE of the Young's modulus is 2.80% and the MRE of the Poisson's ratio is 3.39%.

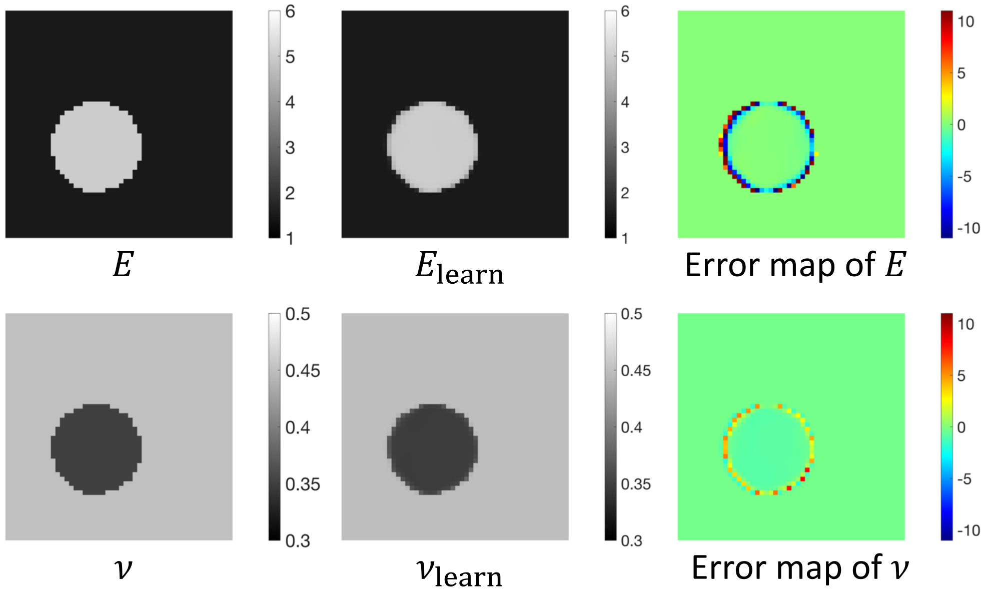

Supplementary Figure 12: ElastNet predictions for the inclusion model in the literature. The inclusion model has a soft background with a circular hard inclusion adopted from Kamali et al[1]. The Young's moduli of the background and inclusion are 1.5 and 5 kilopascal (KPa), respectively. The Poisson's ratios of the background and inclusion are 0.45 and 0.35, respectively. The MRE of the Young's modulus is 0.44% and the MRE of the Poisson's ratio is 0.42%. The accuracies are similar to those reported (0.43% for the Young's modulus and 0.58% for the Poisson's ratio) by Kamali et al., however, ElastNet does not need to know stress distributions on boundaries for elasticity reconstruction.

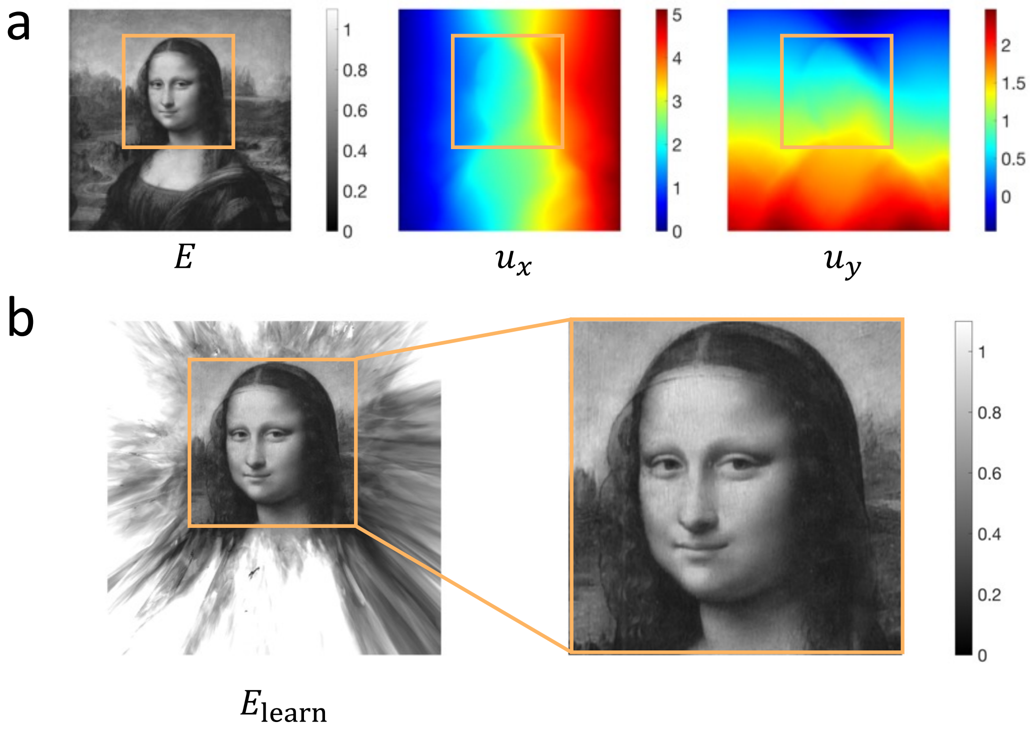

Supplementary Figure 13: ElastNet prediction for the region of interest of the Mona Lisa model. (a) The Young's modulus field (MPa) and displacement field (mm). An average normal strain (ε_{xx}) of 1% is introduced by the displacements applied along the *x*-direction on the boundary. Assuming that we are interested in only the elasticity field in the boxed area. In this case, ElastNet can be trained using the displacement field in the boxed area (256 by 256) to reduce the computational cost compared to using the entire displacement field (512 by 512). (b) The learned Young's modulus (MPa) shows excellent accuracy in the boxed area, with a MRE of 2.16%.

Supplementary Tables

Supplementary Table 1: Prediction accuracy of ElastNet on the Chinese zodiac models.

Number	Name	MRE (%)
1	Rat	2.1012
2	Ox	0.7572
3	Tiger	1.0543
4	Rabbit	1.5273
5	Dragon	4.4199
6	Snake	3.5060
7	Horse	0.7556
8	Goat	1.7978
9	Monkey	2.7284
10	Rooster	2.3117
11	Dog	1.8987
12	Pig	0.6970

Supplementary Table 2: Performances of activation functions.

σ_e	σ_d	MAE
ReLU	ReLU	0.0331
ReLU	Sigmoid	0.0332
ReLU	Swish	0.0059
ReLU	Tanh	0.0119
Sigmoid	ReLU	0.0921
Sigmoid	Sigmoid	0.0924
Sigmoid	Swish	0.0911
Sigmoid	Tanh	0.0929
Swish	ReLU	0.0286
Swish	Sigmoid	0.0332
Swish	Swish	0.0142
Swish	Tanh	0.0220
Tanh	ReLU	0.0293
Tanh	Sigmoid	0.0330
Tanh	Swish	0.0115
Tanh	Tanh	0.0115

Supplementary Notes

Supplementary Note 1: Forward elasticity problems. In this section, we demonstrate the capability of ElastNet to accurately solve forward elasticity problems. In forward elasticity problems, the elasticity field (Young's modulus and Poisson's ratio) and the boundary conditions of an object are given, and the goal is to determine the deformation (displacements or strains) of the object. To evaluate the performance of ElastNet, we use the rose model and apply external displacements of 1% of the model length along the x-direction on the right boundary, resulting in an average normal strain of 1% along the x-direction (ε_{xx}). The boundary conditions, as shown in Supplementary Fig. 1a, include free top and bottom boundaries, and a fixed left boundary. Movements are not allowed along the vertical direction (y-direction) on the right boundary. The boundary conditions are slightly different from those used in the inverse and mixed elasticity problems, as shown in Supplementary Fig. 1b. The left boundary is not fully fixed, and movements are allowed along the vertical direction (y-direction). Movements are also allowed along the vertical direction (y-direction) on the right boundary. ElastNet is not sensitive to the specific boundary conditions, as shown in both this work and our previous work[2]. The displacement field calculated using FEM is shown in Supplementary Fig. 2a and the displacement field predicted using ElastNet is shown in Supplementary Fig. 2b. The R^2 values for the horizontal and vertical components of the displacement field are 0.9999 and 0.9996, respectively, indicating a strong agreement between the two methods. Overall, the results demonstrate that ElastNet can effectively and accurately solve forward elasticity problems.

Supplementary Code

Link to data and code: https://osf.io/bqu4h/

Supplementary Reference

1 Kamali, A., Sarabian, M. & Laksari, K. Elasticity imaging using physics-informed neural networks: Spatial discovery of elastic modulus and Poisson's ratio. *Acta Biomaterialia* (2022).

2 Chen, C.-T. & Gu, G. X. Learning hidden elasticity with deep neural networks. *Proceedings of the National Academy of Sciences* **118**, e2102721118 (2021).
